# Diagnosis-to-surgery lead time and future-health worry in women with primary breast cancer: a longitudinal Swedish registry-based cohort study

**DOI:** 10.1007/s10549-026-08107-z

**Published:** 2026-10-18

**Authors:** Johan Eriksson, Irma Fredriksson, Lena Lindholm, Micael Appelblad, Malin Sund

**Affiliations:** 1 Department of Diagnostics and Intervention/Surgery, Umeå University, Umeå, 901 87 Sweden; 2 Department of Molecular Medicine and Surgery, Karolinska Institutet, Stockholm, Sweden; 3 Department of Breast-, Endocrine Tumors and Sarcoma, Karolinska Comprehensive Cancer Center, Karolinska University Hospital, Stockholm, Sweden; 4 Heart Centre, Norrland University Hospital, Region Västerbotten, Umeå, Sweden; 5 Department of Public Health and Clinical Medicine, Umeå University, Umeå, Sweden; 6 Department of Surgery, University of Helsinki and Helsinki University Hospital, Helsinki, Finland

**Keywords:** Breast cancer, Lead time, Time to surgery, Patient-reported outcome measures, Worry, Quality of life

## Abstract

**Purpose:**

The interval between breast cancer diagnosis and surgery is psychologically demanding, and meeting the Swedish standardised care pathway target has remained challenging. We examined whether diagnosis-to-surgery lead time is associated with patient-reported psychological outcomes.

**Methods:**

We conducted a longitudinal cohort study using Swedish National Quality Register for Breast Cancer data (2020–2024). The cohort comprised 2,906 women with primary invasive breast cancer undergoing surgery, with patient-reported outcome measure (PROM) data at baseline (*n* = 2,906), 6 months (*n* = 2,638), and 12 months (*n* = 2,678). Lead time from biopsy to surgery was modelled continuously (per 1 SD ≈ 19 days). The primary outcome was worry about future health (EORTC QLQ-BR23 item 43); secondary outcomes were general worry, tension, depressed mood, and overall quality of life. Associations were estimated using mixed-effects cumulative link models adjusted for clinical and tumour factors, pandemic era, patient, and healthcare region.

**Results:**

Median lead time was 32 days (IQR 23–42). Longer lead time was associated with greater worry about future health (adjusted OR 1.15 per SD, 95% CI 1.04–1.27), while associations with the secondary outcomes were smaller and not statistically significant. At baseline, high future-health worry was more common with lead times > 35 vs. ≤ 35 days (42.2% vs. 35.1%).

**Conclusion:**

Longer diagnosis-to-surgery lead time was associated with greater future-health worry, with smaller, non-significant associations for general distress and quality of life. The findings highlight the preoperative waiting period as a potential context for improved communication about expected timelines and reasons for delay.

**Supplementary Information:**

The online version contains supplementary material available at https://doi.org/10.1007/s10549-026-08107-z.

## Introduction

Breast cancer is the most common malignancy among women in Sweden, with 8,803 new cases and 1,328 deaths registered in 2024 [1]. Advances in early detection and multidisciplinary treatment have improved survival, and most patients with non-metastatic disease can now expect long-term survival. As survivorship grows, quality of life during and after treatment has become a central outcome, and patient-reported outcome measures (PROMs) capture dimensions not reflected in clinical endpoints alone.

Timely surgery is a cornerstone of breast cancer care. Large registry-based studies suggest that longer diagnosis-to-surgery intervals are associated with worse oncological outcomes, with delays beyond approximately 60 days often cited as a threshold at which survival may be adversely affected [2–4]. In Sweden, the standardised care pathway target for breast cancer surgery was extended from 28 to 35 days in January 2024 to reflect changes in diagnostic practice (replacement of fine-needle cytology with core needle biopsy and increased use of preoperative MRI). The proportion of patients meeting the target has remained below 50%, with 47% operated within the revised 35-day target in 2024 [5]. The pathway interval is measured from well-founded suspicion of cancer and is therefore longer than the biopsy-to-surgery lead time examined here. Importantly, longer lead times do not necessarily indicate suboptimal care; in some cases, they reflect more thorough preoperative work-up.

For patients, the interval between diagnosis and definitive treatment is characterised by uncertainty and anticipatory worry; elevated distress, anxiety, and fear of disease progression have been described in this period [6–8]. However, the independent contribution of lead time to psychological burden remains unclear, as existing studies are methodologically heterogeneous, often single-centre and cross-sectional, and may miss temporal dynamics or cancer-specific worry [9, 10]. National registry data with repeated PROM assessments offer an opportunity to clarify whether lead time relates to patient-reported psychological outcomes at a population level.

Using data from the Swedish National Quality Register for Breast Cancer (NKBC), which collects clinical variables [11] and standardised PROM items [12] at baseline and follow-up, we examined whether lead time from biopsy to primary surgery is associated with worry about future health. Secondary aims were to compare associations across other PROM domains, evaluate linearity of the exposure–outcome relationship, and test robustness in pre-specified sensitivity analyses. To the best of our knowledge, this is the first national registry-based longitudinal study to examine this association using domain-specific PROM data. Understanding whether surgical timeliness is associated not only with oncological outcomes but also with disease-specific patient-reported burden may inform breast cancer pathway evaluation and preoperative communication.

## Materials and methods

### Study design and setting

We conducted a longitudinal cohort study using data from the NKBC, a population-based national quality register with near-complete coverage of incident breast cancer cases in Sweden. The NKBC contains detailed data on patient and tumour characteristics, diagnostics, surgical and oncological treatment, as well as follow-up. PROMs have been collected within the NKBC since 2020 using validated instruments (EORTC QLQ-C30, EORTC QLQ-BR23, EORTC QLQ-LMC21, FACT-ES, and BREAST-Q). Within the NKBC PROM programme, questionnaire content is composed of validated modules from these instruments, with modules administered according to surgical and treatment characteristics. The outcomes analysed in the present study were derived exclusively from the EORTC QLQ-BR23 and QLQ-C30; no outcomes were combined or harmonised across instruments. Baseline PROM implementation varied between reporting units and increased gradually during the study period, resulting in non-random baseline PROM participation. During the implementation phase, distribution of baseline PROMs increased substantially. Among patients receiving a baseline questionnaire, response proportions declined from 35.3% in 2020 to 24.4% in 2024, while registry-level coverage increased from approximately 5% to 13% of all invasive breast cancer cases. Detailed implementation, coverage, and response data are provided in Supplementary Methods. Follow-up questionnaires are delivered via the national 1177.se digital health portal or by post; follow-up coverage exceeds 54% of all invasive cases at 6, 12, and 36 months. The 12- and 36-month follow-ups are sent only to operated patients. The study period was January 2020 to December 2024.

### Study population

Eligible patients were women aged ≥ 18 years with confirmed primary invasive breast cancer who underwent primary breast surgery (breast-conserving surgery or mastectomy) during the study period and had at least one PROM observation. Patients were excluded if they had a prior breast cancer diagnosis, if the recorded lead time was < 1 day (typically when diagnosis was established at surgery) or > 365 days (likely data entry errors or initial treatment refusal), if data on the primary outcome (BR23 item 43) were missing at all time points, or if the tumour was a pure in situ cancer. All included patients were recorded as planned for primary surgery in the NKBC extract; no patients planned for neoadjuvant systemic therapy were present in the analytical cohort.

### Exposure

The primary exposure was lead time from histopathological diagnosis to primary surgery, defined as the number of calendar days from confirmed diagnosis to primary breast surgery as recorded in the NKBC. The diagnosis date corresponds to the date the diagnostic tissue sample (core needle biopsy or, in a minority of cases, fine-needle cytology) was obtained, following the convention of the Swedish Cancer Register. Importantly, this biopsy-to-surgery interval is shorter than the standardised care pathway lead time, which begins at well-founded suspicion of cancer and additionally encompasses the preoperative diagnostic work-up; the 35-day pathway target is therefore not directly applicable to the exposure analysed here. For the primary model, lead time was standardised to a z-score (mean 0, SD 1), so that the estimated effect corresponds to a one-SD increase (≈ 19 days). The 35-day cut-off was used only as a policy-relevant descriptive benchmark and was not used to define the primary exposure or to select an optimal threshold from the data. The primary analysis modelled lead time continuously.

### Outcomes

The primary outcome was the response to EORTC QLQ-BR23 item 43, selected a priori because it directly captures future-oriented health worry, the psychological construct most plausibly related to uncertainty during the diagnosis-to-surgery interval. The item was formulated as: “During the past week – were you worried about your health in the future?”, measured on a four-point ordinal scale (1 = Not at all, 2 = A little, 3 = Quite a lot, 4 = Very much). High worry was defined as response ≥ 3 for descriptive analyses. Secondary outcomes were EORTC QLQ-C30 item 22 (general worry), item 21 (tension), item 24 (depressed mood), and item 30 (overall quality of life; seven-point scale, inverted so higher values consistently indicate worse outcomes).

### Covariates

Covariates were age at diagnosis (continuous), PROM time point (baseline, 6, or 12 months), clinical tumour size (cT1, cT2, cT3+), clinical nodal status (cN0 vs. cN+), histological grade (NHG 1–3, postoperative pathology), surgery type (breast-conserving vs. mastectomy), and biomarker status (ER, HER2, Ki67).

### Statistical analysis

Associations between lead time and each ordinal PROM outcome were estimated using mixed-effects cumulative link models (CLMM; clmm function, R ordinal package), with random intercepts for patient and healthcare region. As the study period coincided with the COVID-19 pandemic, the primary model additionally adjusted for a binary pandemic-era indicator (2020–2021 vs. 2022–2024); a sensitivity analysis replaced the binary indicator with diagnosis year as a five-level categorical variable, and a model without additional calendar-time adjustment was also run for comparison. Three pre-specified sensitivity analyses were conducted: SA1 restricted the cohort to lead times ≤ 90 days; SA2 restricted to cT1 tumours; SA3 was a binary 35-day threshold contrast (≤ 35 vs. > 35 days). Linearity was assessed with a categorical model (≤ 14, 15–21, 22–30, 31–45, > 45 days) and a generalised additive model (GAM) with a cubic regression spline (R package mgcv, k = 4). Models used complete-case data; the potential impact of informative dropout is addressed in the Discussion. Analyses were conducted in R version 4.5.1; statistical significance was defined as a two-sided p-value < 0.05.

### Ethics and reporting

The study was approved by the Swedish Ethical Review Authority (Ref. 2017/463 − 31). Individual patient consent was waived under applicable Swedish law governing registry-based research. Reporting follows the STROBE checklist for observational studies [13]; a completed checklist is provided as a separate supporting file. The cohort comprised women only because the analytical NKBC PROM extract available for this study included women with primary invasive breast cancer; sex was therefore not a primary analytical variable, in line with the SAGER guidelines.

## Results

### Study population

A total of 2,906 patients with primary invasive breast cancer who underwent primary surgery between January 2020 and December 2024 were included. PROM data were available at baseline for all 2,906 patients, at 6 months for 2,638, and at 12 months for 2,678; 82.9% contributed all three time points. Patient characteristics are presented in Table 1.

**Table 1:** Baseline patient characteristics (*n* = 2,906)

Characteristic	*n*	%	Median (IQR)
**Patients, total**			
	2,906		
**PROM observations**			
Baseline	2,906	100%	
6 months	2,638	90.8%	
12 months	2,678	92.2%	
All three time points	2,410	82.9%	
**Age at diagnosis (years)**			
			63 (54–71)
< 50 years	449	15.5%	
50–59 years	715	24.6%	
60–69 years	872	30.0%	
≥ 70 years	870	29.9%	
**Lead time**,** days (diagnosis to surgery)**			
			32 (23–42)
≤ 35 days	1,789	61.6%	
> 35 days	1,117	38.4%	
**Tumour size (clinical T-stage)**			
cT1	2,038	70.1%	
cT2	765	26.3%	
cT3 or higher	103	3.5%	
**Nodal status (clinical N-stage)**			
cN0	2,764	95.1%	
cN+	137	4.7%	
Missing	5	0.2%	
**Histological grade (NHG**,** surgical specimen)**			
Grade 1	666	22.9%	
Grade 2	1,599	55.0%	
Grade 3	518	17.8%	
Missing	123	4.2%	
**Breast surgery type**			
Breast-conserving surgery	2,411	83.0%	
Mastectomy	495	17.0%	
**Axillary surgery**			
Sentinel node biopsy (SNB) only	2,632	90.6%	
Axillary dissection (± SNB)	207	7.1%	
Missing / not recorded	67	2.3%	
**Screen-detected**	1,727	59.4%	
**Healthcare region**			
Stockholm/Gotland	1,154	39.7%	
West	621	21.4%	
North	362	12.5%	
South	301	10.4%	
Southeast	279	9.6%	
Uppsala/Örebro	189	6.5%	

Values are frequencies (%) unless otherwise stated. IQR = interquartile range. NHG = Nottingham Histological Grade (postoperative pathology). cN+ comprised cN1 (*n* = 136), cN2 (*n* = 1), and cN3 (*n* = 0). PROM follow-up counts reflect the number of patients with an observation at each time point

Median age at diagnosis was 63 years (IQR 54–71); median diagnosis-to-surgery lead time was 32 days (IQR 23–42), with 61.6% (*n* = 1,789) operated within ≤ 35 days. Most tumours were T1 (70.1%); 83.0% underwent breast-conserving surgery and 90.6% had sentinel node biopsy only. Detection within the screening programme was recorded for 59.4%. Substantial regional variation was observed: the proportion operated within 35 days ranged from 77.6% in Region North to 56.8% in Region South. Median lead time increased from 25 days (IQR 20–34) in 2020 to 34.5 days (IQR 27–44) in 2024, and the proportion ≤ 35 days declined from 78.1% to 54.8%.

### Primary outcome: worry about future health

Longer lead time was significantly associated with greater worry about future health. Each one-SD increase in lead time (≈ 19 days) was associated with higher odds of reporting greater worry: OR 1.17 (95% CI 1.05–1.29, *p* = 0.003) without additional calendar-time adjustment. In the primary model, additionally adjusted for pandemic era (2020–2021 vs. 2022–2024), the estimate was OR 1.15 (95% CI 1.04–1.27, *p* = 0.008); replacing the binary indicator with diagnosis year as a five-level categorical variable yielded OR 1.13 (95% CI 1.03–1.25, *p* = 0.014). The modest attenuation across progressively stronger calendar-time adjustment (1.17→1.15→1.13) and the lower confidence limit remaining above unity in all three specifications suggest that the observed association was not fully explained by adjustment for calendar-time trends.

For descriptive context, the baseline proportions also differed between the two lead-time groups: 42.2% of patients with lead times > 35 days reported substantial worry about future health, compared with 35.1% among those operated within ≤ 35 days, an absolute difference of 7.1 percentage points (Table 3). The difference was present in all tumour size subgroups and was largest for cT2 tumours (48.6% vs. 37.2%).

### Linearity

The categorical estimates increased across the lower lead-time categories and were similar for 31–45 and > 45 days (Table S2); confidence intervals were wide and overlapping, and the data did not allow a discrete threshold to be identified. The GAM spline yielded edf = 1.67, and model fit was essentially identical to the linear specification (ΔAIC = 0.0), supporting use of lead time as a continuous exposure.

### Secondary outcomes

**Fig. 1:**
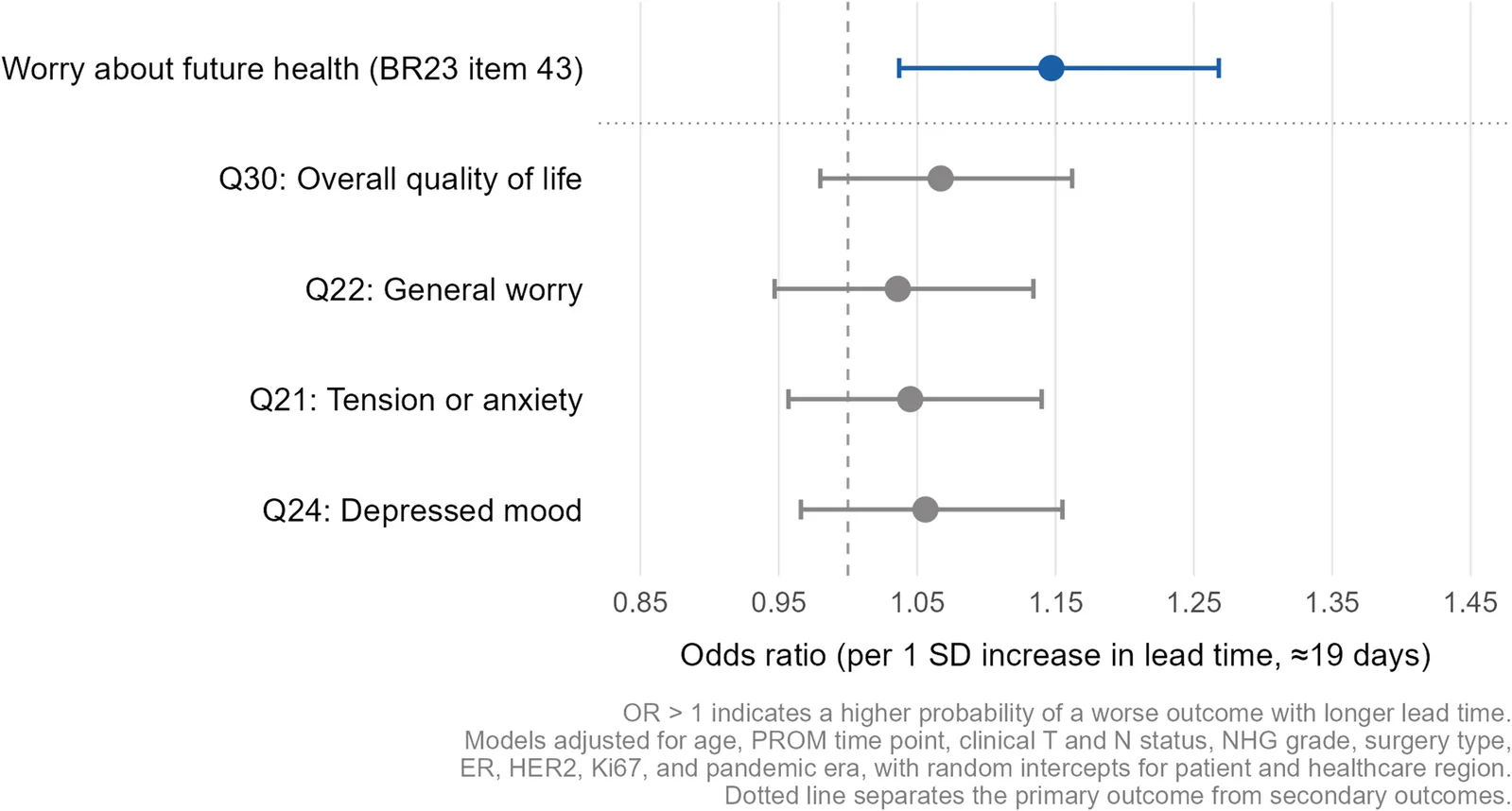
Association between lead time and patient-reported outcome measures in the primary analysis. Odds ratios (OR) per one-SD increase in lead time (≈ 19 days) from mixed-effects cumulative link models, adjusted for age, PROM time point, clinical T and N status, NHG grade, surgery type, ER/HER2/Ki67 status, and pandemic era, with random intercepts for patient and healthcare region. Blue indicates the primary outcome; grey indicates secondary outcomes

In contrast to the primary outcome, none of the four secondary outcomes showed a statistically significant association with lead time (Table 2; Fig. 1). ORs were 1.04 for general worry (Q22; *p* = 0.438), 1.04 for tension (Q21; *p* = 0.330), 1.06 for depressed mood (Q24; *p* = 0.228), and 1.07 for overall quality of life (Q30, inverted; *p* = 0.134). Rates of high scores on tension and depressed mood were similar regardless of lead time (18.2% vs. 18.3% and 18.2% vs. 19.7%, respectively), while the absolute difference for worry about future health was 7.1% points. This pattern was more pronounced for future-health worry than for the secondary outcomes, although the non-significant estimates for the secondary outcomes do not establish absence of association in these domains.

**Table 2:** Association between lead time and PROM outcomes: primary analysis and sensitivity analysis SA1. Odds ratios (OR) per one-SD increase in lead time (≈ 19 days)

Outcome	*n* obs	OR (95% CI)	*p*-value
*Primary analysis (all lead times)*			
Worry about future health (BR23 item 43, primary)	7,457	1.15 (1.04–1.27)	0.008
Q30: Overall quality of life (inverted)	7,743	1.07 (0.98–1.16)	0.134
Q22: General worry	7,367	1.04 (0.95–1.13)	0.438
Q21: Tension or anxiety	7,318	1.04 (0.96–1.14)	0.330
Q24: Depressed mood	7,438	1.06 (0.97–1.16)	0.228
*Sensitivity analysis SA1 (lead time ≤ 90 days)*			
Worry about future health (BR23 item 43, primary)	7,371	1.18 (1.04–1.35)	0.013
Q30: Overall quality of life (inverted)	7,654	1.06 (0.94–1.19)	0.335
Q22: General worry	7,287	1.08 (0.96–1.21)	0.194
Q21: Tension or anxiety	7,237	1.08 (0.97–1.21)	0.168
Q24: Depressed mood	7,355	1.09 (0.98–1.20)	0.112

OR > 1 indicates a higher probability of a worse outcome with longer lead time. Models were adjusted for age, PROM time point, clinical T and N status, NHG grade, surgery type, ER, HER2, Ki67 status, and pandemic era, with random intercepts for patient and healthcare region. SA1 used the same lead-time standardisation as the primary analysis (≈ 19 days per SD)

### Sensitivity analyses

**Fig. 2:**
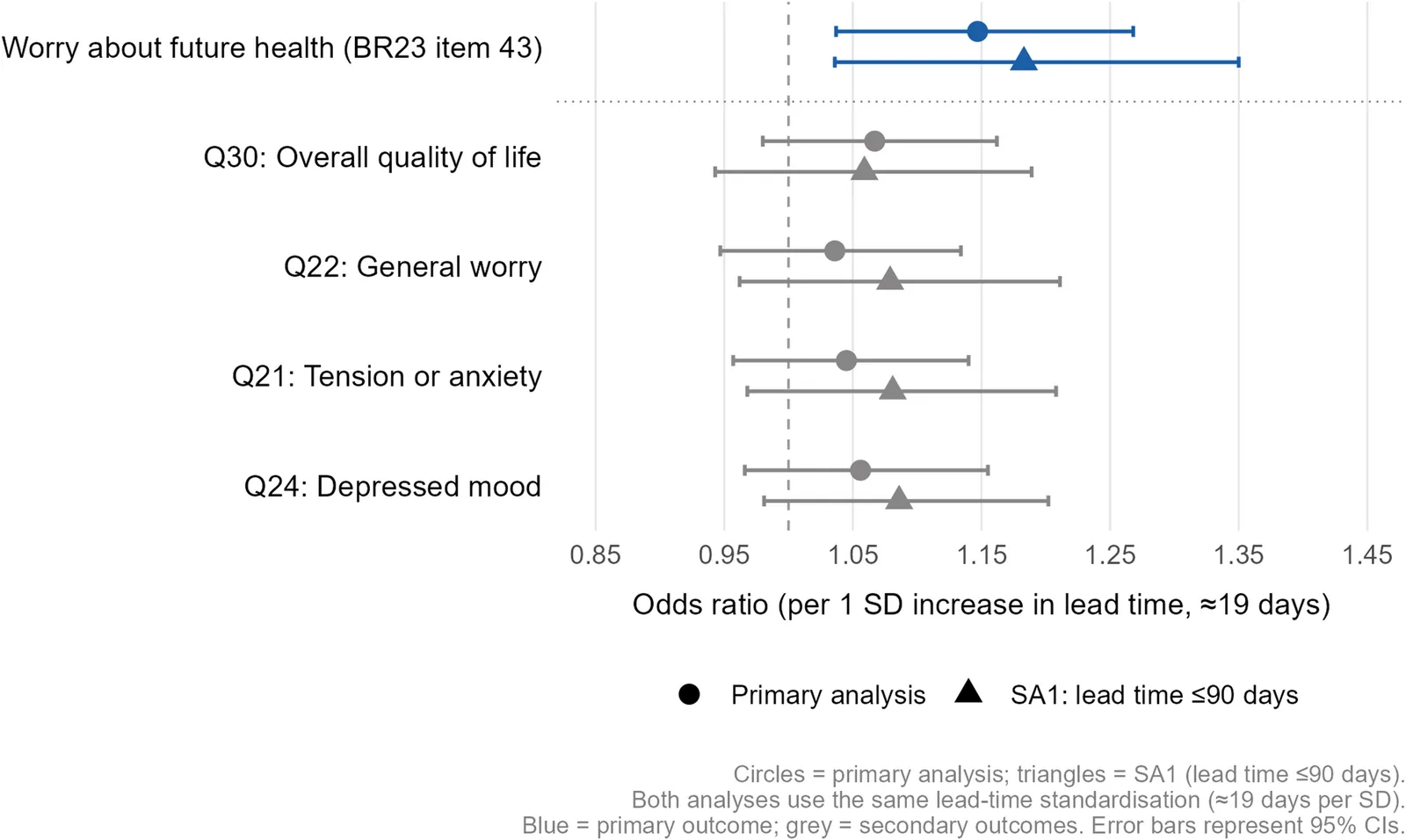
Primary analysis and sensitivity analysis SA1 (restriction to lead times ≤ 90 days). Circles indicate the primary analysis; triangles indicate SA1. Odds ratios are shown per one-SD increase in lead time (≈ 19 days) with 95% confidence intervals. Blue indicates the primary outcome (BR23 item 43); grey indicates secondary outcomes. The dotted line separates the primary outcome from secondary outcomes

When restricted to lead times ≤ 90 days (SA1; *n* = 2,868), the association between lead time and worry about future health remained significant (OR 1.18, 95% CI 1.04–1.35, *p* = 0.013; Table 2; Fig. 2), and all secondary outcomes remained non-significant. In cT1 patients (SA2), the association was not statistically significant (OR 1.10, 95% CI 0.97–1.25, *p* = 0.123), likely reflecting reduced exposure variation: within cT1, 75% of patients in the highest quartile were operated within 50 days (median 43, IQR 36–50). The binary 35-day threshold model (SA3) did not converge due to a singular variance–covariance matrix; therefore, no model-based threshold odds ratio is reported, and inference about the policy cut-off relies on the descriptive contrast in Table 3.

**Table 3:** Proportion reporting high worry about future health (BR23 item 43 ≥ “Quite a lot”) by diagnosis-to-surgery lead time (≤ 35 vs. > 35 days), baseline assessment only

Subgroup	*n* (≤ 35 d)	High worry (≤ 35 d)	*n* (> 35 d)	High worry (> 35 d)
Overall cohort	1,678	35.1%	1,047	42.2%
T1	1,252	34.3%	657	38.8%
T2	387	37.2%	331	48.6%
T3 or higher	39	41.0%	59	44.1%

High worry defined as response ≥ “Quite a lot” on the four-point ordinal scale. Descriptive comparison only; no adjusted model-based estimate for the 35-day threshold was obtained as the binary threshold model (SA3) did not converge

## Discussion

This longitudinal cohort study of 2,906 women with primary invasive breast cancer shows that longer diagnosis-to-surgery lead time is associated with greater worry about future health. Each one-SD increase in lead time (≈ 19 days) was associated with a modest 15% increase in the odds of reporting greater worry in the primary pandemic-era-adjusted model (OR 1.15, 95% CI 1.04–1.27), with consistent estimates across alternative calendar-time adjustments (OR 1.17 without additional calendar-time adjustment; OR 1.13 with diagnosis year as categorical). The association was most evident for future-health worry, whereas estimates for general worry, tension, depressed mood, and overall quality of life were smaller and not statistically significant. This pattern is consistent with, but does not establish, a more specific future-oriented response to longer lead time.

The descriptive contrast provides additional context to the modelling results: at baseline, 42.2% of patients with lead times > 35 days reported substantial worry about future health, compared with 35.1% among those operated within ≤ 35 days. Although the absolute difference was modest and its clinical importance cannot be established from the present study, it may nevertheless be relevant at the population level given the number of patients undergoing breast cancer surgery.

The more pronounced association with future-health worry than with general worry, tension, or depressed mood is compatible with an interpretation involving disease-specific cognitive appraisal. Patients may reason about what a longer timeline implies for prognosis rather than experiencing a uniform increase in distress. This aligns with integrated models of cancer-related anxiety and with conceptualisations of fear of cancer recurrence (FCR), in which future-oriented health worry represents a distinct construct that is particularly responsive to cancer-related cues and prognostic uncertainty [14–17]. Evidence from COVID-19–related treatment changes also linked delay-related experiences to higher recurrence-related worry [18], underscoring the potential value of proactive, health-literacy–sensitive communication during waiting periods.

The observational design does not permit causal conclusions. The findings suggest that diagnosis-to-surgery lead time may be relevant to patient-reported future-health worry in addition to its established clinical importance. The comparatively more pronounced association with future-health worry also raises the possibility that clear preoperative communication about the reasons for, and implications of, waiting time could be valuable, although this was not evaluated in the present study. The continuous model indicates a graded association across the lead-time distribution; because the binary 35-day threshold model (SA3) did not converge, no model-based odds ratio for the policy cut-off is available.

### Strengths

The NKBC provides broad national clinical coverage; PROM analyses draw on a large multi-region sample. The longitudinal design with prospectively collected data from three PROM time points enables repeated-measures modelling accounting for within-patient correlation. Pre-specification of sensitivity analyses and secondary outcomes reduces the risk of selective reporting. The inclusion of several conceptually related PROM outcomes enabled comparison of the magnitude and precision of associations across different dimensions of psychological well-being. The linear specification was supported by complementary categorical and spline-based analyses.

### Limitations

Several limitations should be acknowledged. First, the observational design precludes causal inference, and residual confounding by unmeasured or incompletely captured disease severity factors cannot be excluded despite adjustment for clinical T and N status, NHG grade, and tumour biology (ER, HER2, Ki67). Patients with more complex disease may both wait longer and worry more; the SA1 restriction to lead times ≤ 90 days yielded a directionally consistent and significant result (OR 1.18, 95% CI 1.04–1.35), supporting robustness within the clinically most relevant range.

Second, treatment sequence was handled using the planned-treatment variable in the NKBC extract; all included patients were classified as planned for primary surgery, but treatment-sequence misclassification cannot be entirely excluded in registry data. All included patients were planned for primary surgery, and surgery type (breast-conserving surgery versus mastectomy) was included in the adjusted models. Definitive decisions regarding adjuvant systemic treatment and radiotherapy are generally made postoperatively in this treatment sequence. Detailed data on subsequent chemotherapy, endocrine therapy, and radiotherapy were not available in the analytical extract and could therefore not be included in the models. These subsequent treatments may nevertheless influence future-health worry at the 6- and 12-month assessments.

Third, the study period coincided with the COVID-19 pandemic, during which case mix and population-level health-related worry may have differed from later years. The primary analysis adjusted for a binary pandemic-era indicator, and a sensitivity analysis used diagnosis year as a five-level covariate. Adjusted estimates were attenuated only modestly (1.17→1.15→1.13), and the lower confidence limit remained above unity throughout, suggesting that the observed association was not fully explained by adjustment for calendar-time trends. Of note, the BR23 item does not distinguish cancer-specific worry from generalised health anxiety, a limitation particularly relevant during periods of widespread health-related distress.

Fourth, PROM participation in the NKBC is non-random at two levels. At the unit level, baseline PROM implementation and resulting population coverage were low and heterogeneous across the study period (approximately 5% of all invasive cases in 2020 and 13% in 2024). Units with more systematic PROM implementation may differ not only in patient case mix but also in organisational practices, including patient information and waiting-time management, which could influence both lead time and reported worry. Within the analytical cohort, item-level non-response was low (BR23 item 43 was answered by 93.8–97.5% of respondents at each time point) and follow-up retention was high (82.9% contributed all three time points); baseline characteristics of patients contributing all three time points were similar to those contributing fewer observations, although dropout related to unmeasured factors or outcomes cannot be excluded. However, the available analytical extract did not permit a direct comparison between PROM participants and otherwise eligible patients who did not contribute PROM data; selection at entry into the PROM cohort therefore remains an important limitation. Previous population-based cancer studies have shown that PROM or survey participants may differ systematically from non-participants in sociodemographic and clinical characteristics, disease severity, and survival, indicating that selection into such cohorts can affect generalisability [19, 20]. Accordingly, selection bias at entry into the present PROM cohort cannot be excluded. Because baseline PROM implementation was incomplete, absence of a baseline PROM could reflect either non-distribution of the questionnaire or non-response after distribution. The analytical extract did not contain invitation data for the full eligible population, and these mechanisms could therefore not be distinguished directly. Selective administration of PROMs, for example to patients perceived as more able or willing to complete questionnaires, cannot be excluded. The magnitude and direction of any resulting selection bias are uncertain. Fifth, the T1-restricted analysis (SA2) was non-significant, likely reflecting reduced exposure variation. Sixth, the binary 35-day threshold model (SA3) did not converge, so inference about the policy cut-off relies on descriptive contrasts.

Seventh, baseline PROMs are intended to be completed in connection with the diagnosis disclosure and before treatment, preferably as close to diagnosis as possible. However, the exact completion date was not available in the analytical extract. We could therefore not determine how much of the subsequently observed biopsy-to-surgery interval had elapsed when baseline future-health worry was reported. Accordingly, the baseline association should not be interpreted as the effect of having experienced the full waiting interval.

## Conclusions

Longer diagnosis-to-surgery lead time was associated with greater patient-reported worry about future health, while associations with general distress, depressed mood, and overall quality of life were smaller and not statistically significant. The comparatively more pronounced association with future-health worry is consistent with, but does not establish, a more specific future-oriented response. Although the observed association was modest and cannot establish causality, the findings highlight the potential relevance of waiting time and preoperative communication to patients’ future-health worry.

## Supplementary Information

Below is the link to the electronic supplementary material.

Supplementary Material 1

## Data Availability

Data from the NKBC are available on reasonable request, subject to approval by the Swedish Ethical Review Authority and the NKBC register holder. Restrictions apply to the availability of these data, which were used under licence for this study.
