# Supplementary material for: Diagnosis-to-surgery lead time and future-health worry in women with primary breast cancer: a longitudinal Swedish registry-based cohort study
Source: Breast Cancer Res Treat. 2026 Oct 18;220(1):3. doi: 10.1007/s10549-026-08107-z (PMC13677628; doi:10.1007/s10549-026-08107-z)
Supplement: Supplementary file 1 [file 10549_2026_8107_MOESM1_ESM.docx]

**Supplementary Material**

*Diagnosis-to-surgery lead time and future-health worry in women with primary breast cancer: a longitudinal Swedish registry-based cohort study*

**Supplementary Methods**

***S1. PROM coverage and response rates***

Patient-reported outcome measures (PROMs) have been collected within the Swedish National Quality Register for Breast Cancer (NKBC) since 2020 via the INCA platform. The implementation has been gradual and has differed across reporting units and over time.

Among patients who received a baseline PROM questionnaire, the proportion completing the questionnaire was 35.3% in 2020 and 24.4% in 2024. Despite this decline in response among recipients, increased distribution resulted in registry-level baseline PROM coverage increasing from approximately 5% of all incident invasive breast cancer cases in 2020 to approximately 13% in 2024.

Baseline PROM coverage was substantially affected by the COVID-19 pandemic during 2020 and 2021, when register-administrative routines were disrupted. Coverage has improved over the study period: estimated baseline PROM coverage of all incident invasive breast cancer cases was approximately 5% in 2020, rising to approximately 13% in 2024. The reasons for low and heterogeneous baseline coverage are multiple, including: (i) site-level differences in PROM workflow integration, (ii) variation in whether the baseline questionnaire was offered before or only after the diagnosis-disclosure visit, and (iii) the technical migration of distribution mechanisms between paper and the national 1177.se digital health portal.

Follow-up PROM coverage is higher than baseline coverage. According to the NKBC annual reports, follow-up PROM coverage exceeds 54% of all invasive breast cancer cases at 6, 12, and 36 months after diagnosis. The 12- and 36-month follow-ups are issued only to patients who have undergone primary surgery.

Within the analytical cohort (i.e., patients who responded to at least one PROM assessment and met all other inclusion criteria), follow-up retention was high: 90.8% of the 2,906 patients who answered at baseline also answered at 6 months, 92.2% at 12 months, and 82.9% answered at all three time points (Supplementary Table S1).

Item-level non-response on the primary outcome (EORTC QLQ-BR23 item 43) was low among patients who participated in a given PROM round: 93.8% to 97.5% answered the item at each time point. Models used complete-case data; the potential impact of informative dropout is addressed in the main manuscript Discussion.

***S2. Linearity assessment of the lead-time exposure***

The linear specification of lead time used in the primary model was assessed using two complementary approaches.

First, a categorical model was fitted with five lead-time categories (≤14, 15–21, 22–30, 31–45, and >45 days), with ≤14 days as the reference category. Point estimates were above 1.0 in all categories, with the highest estimate observed for 31–45 days (OR 1.62, 95% CI 1.01–2.61). The categorical estimates did not indicate a clear discrete threshold (Table S2).

Second, a generalized additive model (GAM) was fitted using a cubic regression spline for lead time (mgcv package, k=4). The estimated effective degrees of freedom (edf) was 1.67. Model fit was essentially identical to that of the linear specification (ΔAIC=0.0), providing no evidence that the spline specification improved model fit. These findings support the use of lead time as a continuous exposure in the primary analysis.

***S3. Sensitivity analyses: detailed specifications***

Three pre-specified sensitivity analyses were performed:

**SA1 (cohort restriction):** Restricted to patients with lead time ≤90 days (n=2,868) to reduce influence of outliers and clinically unusual delays. The primary association remained significant (OR 1.18, 95% CI 1.04–1.35, p=0.013).

**SA2 (T1 restriction):** Restricted to clinical T1 tumours (n=2,038), the most prevalent subgroup. Within T1, exposure variation was reduced: 75% of patients in the highest quartile were operated within 50 days (median 43, IQR 36–50). The primary association was non-significant (OR 1.10, 95% CI 0.97–1.25, p=0.123), likely reflecting reduced statistical power and reduced exposure range.

**SA3 (binary 35-day threshold):** Binary contrast (≤35 vs >35 days). The model did not converge, returning a singular variance–covariance matrix. We attempted re-fits with simplified random effects structures (random intercept for patient only; random intercept for healthcare region only) without resolution. We therefore do not report a model-based threshold odds ratio. Inference about the policy-relevant 35-day cut-off is based on the descriptive contrast presented in Table 3 of the main manuscript.

SA1 and SA2 used the same lead-time standardisation as the primary analysis (approximately 19 days per SD).

**Supplementary Tables**

**Table S1.** PROM completion by time point in the analytical cohort (n=2,906).

| **Time point** | **n responders** | **% of cohort** | **BR23 Q43 % answered** |
| --- | --- | --- | --- |
| Baseline (pre-treatment) | 2,906 | 100.0% | 97.5% |
| 6 months | 2,638 | 90.8% | 94.2% |
| 12 months | 2,678 | 92.2% | 93.8% |
| All three time points | 2,410 | 82.9% | *–* |

*BR23 Q43 = EORTC QLQ-BR23 item 43 ("During the past week – were you worried about your health in the future?"). Item-level response rates are the proportion of PROM responders at each time point who answered Q43.*

**Table S2.** Categorical assessment of the association between diagnosis-to-surgery lead time and future-health worry.

| **Lead-time category** | **OR** | **95% CI** | **p-value** |
| --- | --- | --- | --- |
| ≤14 days | Reference | – | – |
| 15–21 days | 1.18 | 0.71–1.97 | 0.523 |
| 22–30 days | 1.28 | 0.79–2.07 | 0.309 |
| 31–45 days | 1.62 | 1.01–2.61 | 0.047 |
| >45 days | 1.60 | 0.97–2.65 | 0.068 |

*Odds ratios are from a mixed-effects cumulative link model with ≤14 days as the reference category, adjusted for PROM time point, age, clinical T stage, clinical N stage, histological grade, breast surgery type, ER status, HER2 status, Ki67 status, and pandemic era, with random intercepts for patient and healthcare region.*
